# Mucin immobilization in calcium alginate: A possible mucus mimetic tool for evaluating mucoadhesion and retention of flavour

**DOI:** 10.1016/j.ijbiomac.2019.07.148

**Published:** 2019-10-01

**Authors:** Vlad Dinu, Gleb E. Yakubov, Mui Lim, Katherine Hurst, Gary G. Adams, Stephen E. Harding, Ian D. Fisk

**Affiliations:** a National Centre for Macromolecular Hydrodynamics, School of Biosciences, University of Nottingham, Sutton Bonington Campus, Leicestershire, UK; b Division of Nutrition, Food and Dietetics, School of Biosciences, University of Nottingham, Sutton Bonington Campus, Leicestershire, UK; c School of Health Sciences, Faculty of Medicine and Health Sciences, Queen's Medical Centre, Clifton Boulevard, Nottingham, UK; d Universitetet i Oslo, Postboks 6762, St. Olavs plass, 0130 Oslo, Norway

**Keywords:** Mucus biomimetic, Flavour

## Abstract

To reduce animal testing, there is a need to develop novel *in-vitro* models for evaluating the retention of bioactive compounds in food and pharmaceutical products. Here, a mucus-mimetic platform was developed through a one-step approach based on encapsulating mucin within alginate gel beads. We found that mucins form micron sized aggregates distributed across the surface of the calcium-alginate bead, as shown by environmental scanning electron microscopy (ESEM). Retention of bioactive compounds on the mucin-functionalised surface was tested using a commercial orange drink formulation. To aid flavour retention, different mucoadhesive polymers with varying charge, including anionic, neutral and strongly cationic, were tested for their ability to interact with mucin and aid retaining flavour compounds within the mucin-alginate bead. The alginate-mucin mucus mimic was validated using an *ex-vivo* bovine tongue, with the flavour retention results showing qualitative agreement. The developed method proved to be a convenient, efficient tool for providing information on the effectiveness of mucoadhesive polymers without variability, safety and sustainability issues associated with an *ex-vivo* or *in-vivo* system. We propose that by encapsulating other relevant oral proteins, alongside mucins, current gaps between *in-vitro* and the *ex-vivo* systems may be narrowed.

## 1 Introduction

During new product development or reformulation, food and pharmaceutical companies need to address the release and retention properties of the product onto target tissues, such as the mouth, skin or gastrointestinal (GI) tract. At the moment, a large number of food products are being tested on animal tissues. It was reported that over 60% of scientific research in the area of mucoadhesion involve the use of laboratory animals, specifically raised for their mucosal surfaces [1,11]. However, the current trend, supported by sustainability campaigns and animal rights organizations, is to discourage industry and academia from performing testing on animal tissues and aim to use more sustainable options.

Currently, an alternative is to use byproducts resulting directly from the meat industry. Although discarded animal tissues from abattoirs provide closer representations of *in-vivo* mucosa, there are still a number of issues associated with their use, such as limited shelf life and increased risk of cross contamination [2,3], making them less than ideal for assessing the retention of bioactive compounds in foods, oral care and E-liquid products. Several new alternatives have been proposed to overcome those limitations [[4], [5], [6]]. Some innovations include the development of mammalian epithelial cell lines grown on collagen membranes, which resemble the surface morphology of mucus [7,8]. However, the lengthy and costly nature of such models combined with the low yield of the final product limits its potential industrial and development applications [9,10]. A summary of some of the more recent developments in mucosa-mimics along with a number of mucoadhesion assays has been reviewed by [11].

More accessible approaches make use of commercially available mucin powders, isolated and purified from the bovine or porcine GI tract. Since mucin glycoproteins represent the primary macromolecular constituent of mucus, their use enables the development of a wide array of model systems, from aqueous solutions to partially hydrated films, or grafted onto cellulose and other biomimetic polymer substrate [[12], [13], [14], [15]]. Alternative approaches moved away from the conventional mucus/mucin-based systems and developed alternative methods based on hydrocolloids and/or synthetic polymers. For instance, cellulose, locust bean gum gels or carbopol microgels have all been employed as surrogates for bioadhesion assays by mimicking the rheological and adhesive properties of mucus [16,17]. For example, the use of 2 hydroxyethyl methacrylate (HEMA) cross linked with sorbitol methacrylate (SMA) monomers and N-acryloyl glucosamine (AGA) were shown to possess carbohydrate-like rheology of mucus [17].

An appropriate model surface is critical for enabling accurate predictions of the mucoadhesive properties of polymers. For instance, Cook et al. (2015) analysed an *ex-vivo* system based on porcine oral tissue in order to analyse the mucoadhesive strength of three biopolymers: sodium alginate, sodium carboxymethyl-cellulose (CMC) and pectin (DE < 30). They previously suggested that pectin was by far the most potent in its ability to enhance the retention of sodium *in-vitro*, but the *ex-vivo* tongue experiments confirmed that CMC was the more potent in increasing salt bioavailability. By contrast, *in-vivo* sensory results recently established that CMC can reduce sodium perception, despite scoring high for attributes such as adhesion and mouth-coating [18,19]. Such disagreements highlight the importance of selecting a suitable model system for evaluating mucoadhesion.

Some *in-vivo* studies may still provide a clearer picture, for example Atmospheric Pressure Chemical Ionization- Mass Spectrometry coupled with the MS-Nose interface can enable the retronasal quantification of volatile compounds present in the oral cavity, but can be very challenging due to the lack of compound specificity, or the very weak signals detected from real foods [20]. Before consumer phases, *in-vivo* and *ex-vivo* animal testing still remain the primary means of evaluating product interactions.

Perception of flavour during oral processing is ultimately determined by the rate of release of aroma and taste compounds from the salivary bolus, towards the aroma and taste receptors, before the food or medicine is ingested. Otherwise, flavour, along with salt or other bioactive compounds, is rapidly lost through ingestion and is therefore not available for perception. The common strategy to combat the loss of sensory attributes is to increase the concentration of flavour and taste compounds in food, leading to nutritionally compromised products with high sodium, sugar and excessive levels of flavourings. To combat this issue, there is a need to develop safe and effective mucoadhesive biopolymers capable of extending residence time and promoting the delivery of flavour compounds in the oral cavity.

In this study, we developed a rapid and sustainable biomimetic alternative for evaluating the retention of flavour compounds in the presence of different polymer mucoadhesives. Physiological concentrations of mucin were encapsulated in calcium alginate spheres of approximately four millimeters in diameter using the well-established ion exchange reaction [21,22]. It is hypothesized that mucin glycoproteins are anchored along the surface of the calcium alginate complex, thus influencing the chemistry of the system. To validate the efficiency of the *in-vitro* model, the surface of an *ex-vivo* bovine tongue was used to assess the ability of mucoadhesive excipients to aid the retention of bioactive flavour compounds. Environmental scanning electron microscopy (ESEM) and Dynamic Light Scattering were used to examine the surface morphology and surface chemistry of the beads. Gas Chromatography-Mass Spectrometry (GC–MS) was used along with conductivity analysis to measure the release of the aroma and salt compounds.

## 2 Results and discussion

### 2.1 Characterization of mucin-alginate beads

**Fig. 1:**
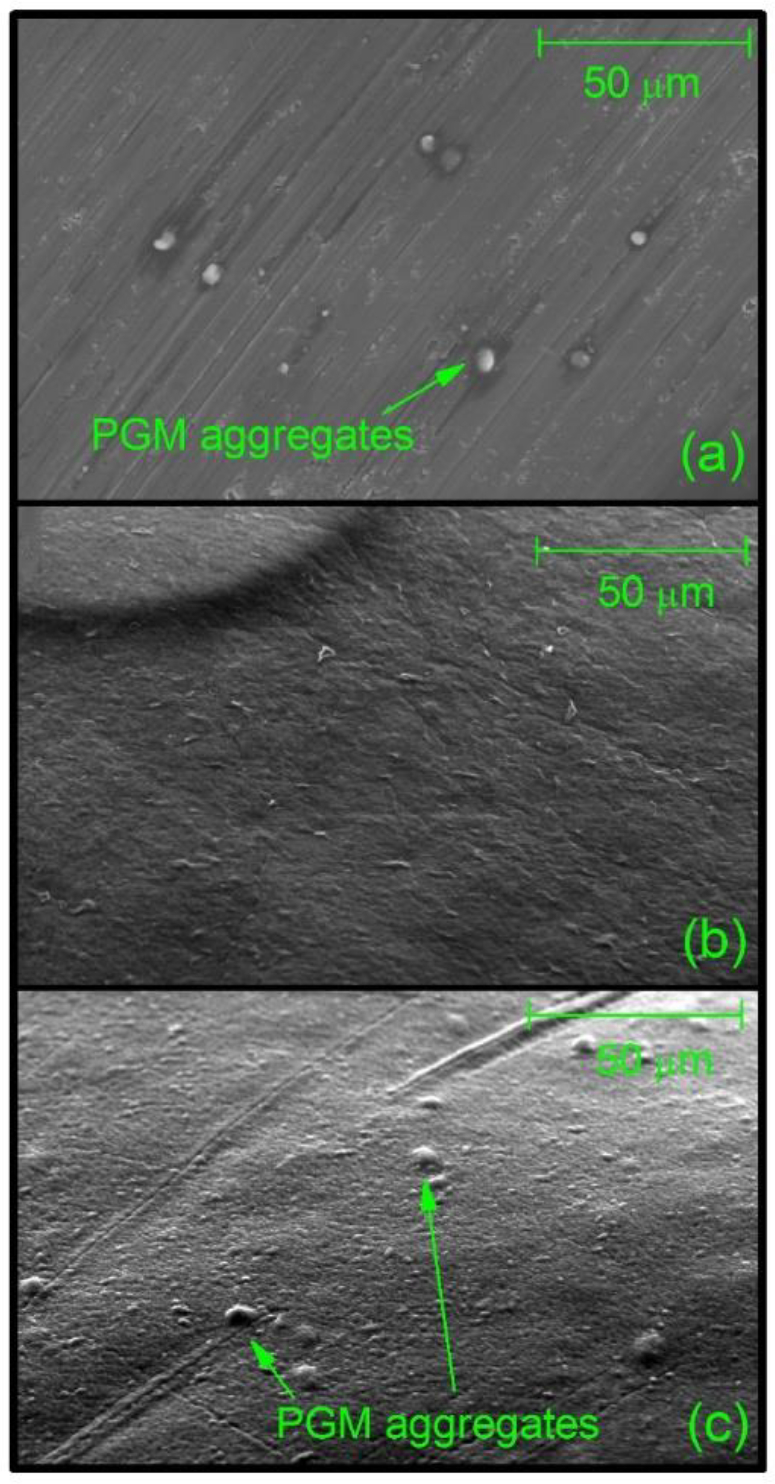
Environmental scanning electron micrographs of pig gastric mucin (PGM) suspended in 0.1 M PBS, pH 7.0 (a), surface alginate bead (b) and the surface of the alginate-mucin bead (c).

It is hypothesized that entrapment of mucin in alginate beads would result in a mucosa mimetic surface using the chemical and physical properties of mucins. Encapsulation of mucin in calcium alginate was used to produce millimeter-sized beads with a mucin-functionalized surface. The beads were subjected to controlled dehydration in the ESEM sample chamber at operating pressures ranging from ~4 to ~5 Torr. The surface of the alginate-mucin was visualized and compared to the control beads, containing no mucin. Mucin control revealed the formation of larger well-separated globular aggregates (<1 μm) present on the streaky surface of the metal sample holder (Fig. 1a). Similar globular features are visible on the surface of the mucin-alginate bead (Fig. 1c). By contrast, the surface of the control alginate bead appeared smoother, without the presence of large aggregates (Fig. 1b).

The apparent zeta potential was measured to assess changes in the surface charge of the mucin-alginate bead. Although it was not possible to measure the actual charge, due to the presence of high Ca^2+^ concentrations contributing to overestimates, our values indicate a relative decrease in the zeta potential from 19.5 mV (±2.9) to −0.5 mV (±1.3), after loading with mucin. This is thought to be attributed to the entrapment of mucin within the gelled alginate matrix, which is consistent with ESEM results.

Four different biopolymers were investigated: anionic CMC, neutral non-ionic pullulan, weakly cationic dimethylaminoethyl (DMAE) pullulan and strongly cationic chitosan (70% de-acetylation). All polymers had a weight average molecular weight of approximately 200,000 g/mol. The mucin-alginate beads, along with the control beads (alginate only) were immersed in the different biopolymer formulations.

**Fig. 2:**
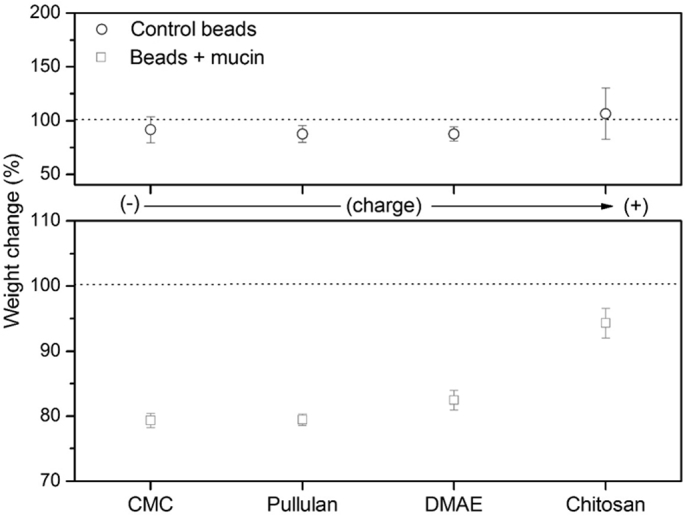
Changes in the weight of control and mucin beads after immersion in different biopolymer formulations for 30 min. Weight change is directly proportional to changes in the hydrated radius of the spheres. Values are expressed as mean ± SD (*n* = 3).

Changes in the weight of the beads were measured for each treatment and plotted in the order of polymer charge (Fig. 2). Within the experimental error, there were no differences between different polymer treatments in the control beads, although chitosan led to the largest apparent weight change, which we hypothesized is due to hydration.

By contrast, different biopolymer excipients had a pronounced effect on the weight and size of mucin-alginate beads. All treatments led to a marked decrease the level of hydration of the beads, attributed to ion exchange reactions in the presence of the low ionic strength formulation, such as the migration of calcium ions from the bead. The magnitude of the observed hydration effect was found to be dependent on the net charge of the polymer. For instance, the size of the beads immersed in anionic CMC and neutral pullulan appeared smallest, as opposed to the beads immersed in cationic polymers, which were significantly heavier (Fig. 2).

**Fig. 3:**
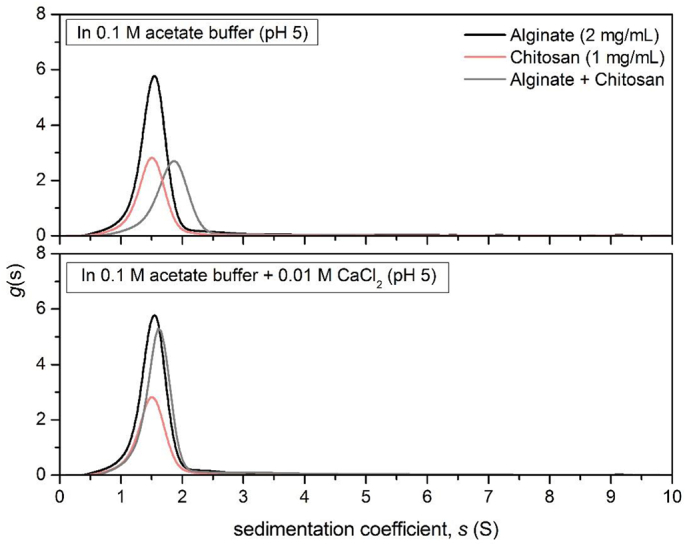
Sedimentation velocity, *g*(s) analysis showing the sedimentation coefficient distributions of alginate (2.0 mg/mL) and the result of its interactions with chitosan (1 mg/mL) in the presence and absence of 0.01 M CaCl_2_. Rotor speed: 40000 rpm (120,000 g), 20.0 °C.

Based on the current hypothesis, this effect is attributed to strong electrostatic interactions between the charged polymer groups and the negatively charged mucins, *via* their sialic acids or sulfate protein groups. However alginate mannuronic or guluronic acid groups can also interact with chitosan, but the extent of the interaction with alginate is reduced by the presence of calcium ions. This has also been confirmed by an SV-AUC experiment, in which we investigated the effect of calcium chloride (CaCl_2_) on the alginate chitosan interaction (Fig. 3).

In the absence of CaCl_2_, the addition of 1 mg/mL chitosan to a 2 mg/mL alginate solution lead to the depletion the alginate concentration and the formation of large supramolecular aggregates of over 200 Svedberg (Fig. 3 top). By contrast, the presence of 0.01 M CaCl_2_ appeared to significantly prevent the alginate-chitosan interaction, although a small proportion of the alginate concentration was decreased (Fig. 3 bottom). The reason for the very low calcium chloride concentrations used was to avoid the formation of hard alginate gels in the AUC centerpieces. In reality, higher CaCl_2_ concentrations would have an even stronger shielding effect. Therefore, in the current study, it is implied that interactions with mucin are the main contributors to the differences observed in the hydration of the biomimetic beads.

### 2.2 Retention of aroma and taste compounds

The biopolymers were tested in the presence of a real food system, Robinson's orange squash, prepared according to the manufacturer. However, hexanal, octanal and decanal were also added to the commercial product, in order to address interactions with linear aldehydes.

**Fig. 4:**
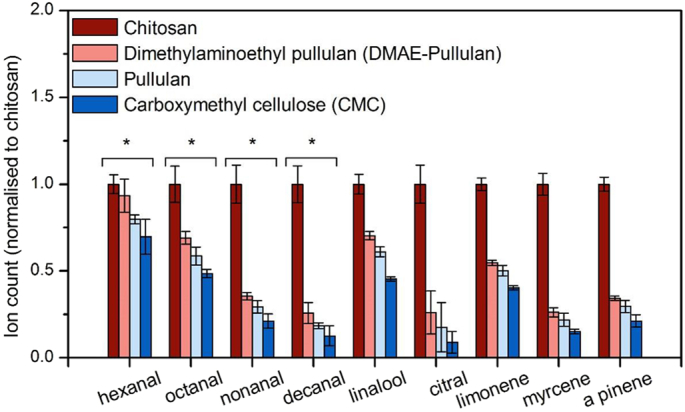
GC–MS results showing the relative volatile intensity after immersion in different biopolymer solutions. Aroma compounds are naturally found in the orange squash fomulation while the linear aldehydes were additionally added. The comparison is made by Tukey's *post hoc* test to calculate the *P*-values (*P* < 0.05*). The data are shown as mean ± SD, *n* = 3.

There was a direct effect of each biopolymer on each aroma compound, although this effect varied in intensity, for example the strongly cationic chitosan retained significantly higher concentrations of volatile compounds (Fig. 4), when compared to the other biopolymers. An effect was also observed for linear aldehydes, showing a hydrophobicity (log*P*) dependent effect, where they decrease in intensity with increasing hydrophobicity. It is also worth mentioning that of the compounds investigated, limonene, nonanal and linalool are the most abundant aroma compounds present in the orange squash, and correspond to over 80% of all volatiles present.

Significant differences (*p* < 0.01) were observed in the retention of volatile aroma compounds between the oppositely charged ionic polymers, *i.e.* chitosan and CMC, DMAE-pullulan and CMC (*p* < 0.05). By contrast, with the exception of linalool, limonene and myrcene, no significant differences were observed between CMC and pullulan (p < 0.05), although the relative intensity increased by an average of ~10% in the presence of pullulan (Fig. 4). However, no changes in weight were observed for the two treatments (Fig. 4). This may be due to different physiochemical properties between the CMC and pullulan, such as branching and higher viscosity for CMC, although there were no differences in the viscosity and pH of the dilute orange drinks.

Additionally, we performed an experiment to quantify the total acidity contained within the model food (Table 1). This was performed by exhaustively titrating the liquid drinks until they reach a pH of ~8 (Eq. 1). Although the pH of the different orange drink formulations was virtually identical, approximately 4.5, small differences were found in the chitosan formulation, attributed to the additional presence of acetic acid used in the solubilisation of chitosan. Therefore, the slightly higher acidity may also contribute to the higher partitioning of aroma compounds [23,24].

**Table 1:** Titratable acidity (TA) of final orange drinks containing different polymer formulations (error based on ±50 μl 0.1 N NaOH).

Biopolymer	TA (g/100 mL)
CMC	0.34(±0.01)
Pullulan	0.33(±0.01)
DMAE-pullulan	0.33(±0.01)
Chitosan	0.40–0.43 (±0.01)*

*Depending on main acid present *i.e.* citric (eq. factor 0.064) or acetic (eq. factor 0.060).

**Fig. 5:**
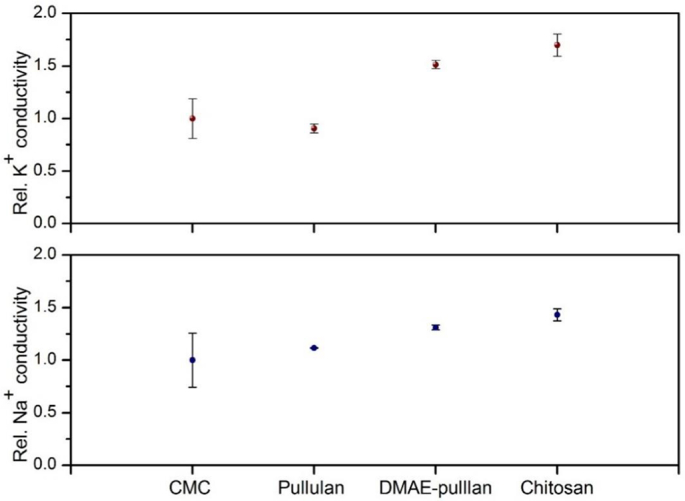
Conductivity results showing the relative Na^+^ and K^+^ concentration on the mucin beads after immersion in different biopolymer solutions. Values are determined by subtracting the values of the control beads. Values are expressed as mean ± SD (n = 3).

In an analogous way, the ability of our *in-vitro* surface to retain sodium and potassium was investigated (Fig. 5). The results appear to be in complete agreement to our hypothesis, despite the different interaction mechanism of ions with mucin or other ionic polysaccharides present in the system [25]. This includes charge shielding of carboxylic acids, sialic acid and reduction in polymer viscosity. Although a similar trend was observed, changes in relative intensity of sodium ions retained in the presence of CMC were not significantly different, possibly due to the small sample size, or *via* any of the mechanisms mentioned previously.

By contrast to anionic polymers, salt retention in the presence of cationic polymers increased by up to 50%, reinforcing the strong effect of electrostatic interactions in mucoadhesion (Fig. 5). It may be possible sodium retention is related to changes in hydration but also to competition with calcium ions. Another possibility may result from the anionic polymer effect, previously shown to stunt the perception of sodium by reversibly serving as the anion associated twitho the Na^+^ ions [26].

### 2.3 Comparison to the *ex-vivo* bovine tongue surface

**Fig. 6:**
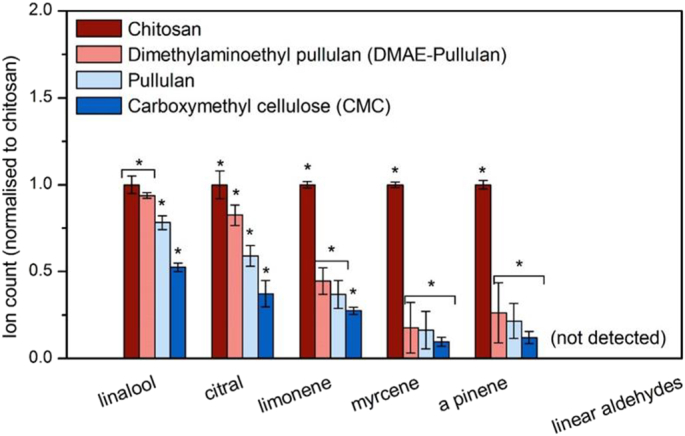
GC–MS results showing the relative volatile retention on the mucin beads after immersion in different biopolymer solutions. The comparison is made by Tukey's *post hoc* test to calculate the *P*-values (*P* < 0.05*). The data are shown as mean ± SD, n = 3.

We compared the retention of volatile aroma compounds on the *in-vitro* system with the oral *ex-vivo* tissue (Fig. 6). The model used in our study was made from the dorsal layer of a fresh bovine tongue. Results appeared to be in full agreement with our *in-vitro* findings, with volatile retention being directly correlated to the charge of the polymers, However, major differences are observed for the added linear aldehydes, which were not detected by the GC. Other studies have reported this effect, suggested to arise from the formation of irreversible covalent bonds between linear aldehydes and amino acids, such as lysine [27].

### 2.4 General discussion

Previous *ex-vivo* experiments using real human saliva have also confirmed a significant reduction in the headspace concentration of linear aldehydes, but not to this extent [28]. It is worth highlighting potential issues associated with the validity of this and other similar *ex-vivo* tissues [29]. It is possible that the excised tissue may not be the most accurate representation of an oral surface after all, due to the higher proportion of exposed meat tissue on the ventral surface of the tongue, uncovered by a mucus layer. This may be an explanation for the loss in the linear aldehyde signal, compounds well known to irreversibly bind to protein groups, which apart from the ‘naked’ regions present sparsely present in mucus, are absent in the *in-vitro* model.

However, other proteins or enzymes can be encapsulated to provide a more accurate representation of the target tissue. Another advantage is that the gel structures can be tailored to meet specific applications, by varying the concentrations of calcium chloride or sodium alginate. Additionally, the gelled alginate beads can be adjusted in size or molded into completely different shapes, such as thin sheets, depending on the test needed to assess for polymer mucoadhesion.

## 3 Conclusion

Given the anionic properties of mucins, electrostatic interactions with polymers play a key role in mucoadhesion, along with hydrogen bonds and van der Waals forces of attraction [11]. Here, we took advantage of some of these functional electrochemical properties to validate a model *in-vitro* surface for its ability to retain flavour compounds as a function of *co*-ingredient polymer mucoadhesives. The *in-vitro* mucus mimic is synthetized using the ion exchange calcium-alginate reaction, which led to the formation of mucins immobilised in calcium alginate. Electron microscopy and Zeta potential results indicated that mucins and mucin aggregates impart their characteristic anionic properties to the surface of the beads. The *in-vitro* tool was proven able to retain flavour compounds, naturally present in orange squash formulations in a similar way to a bovine tongue surface, confirming the electrostatic theory. However this excluded linear aldehydes which are suggested to bind to the dorsal part of the tongue, contaminated by excision. Moreover, the immobilization of other relevant proteins may further improve the system and ultimately reduce future needs to use animal based tissues.

## 4 Materials and methods

### 4.1 Materials

Stock solutions of pig gastric mucin (Sigma-Aldrich, M1778, type III) and sodium alginate (Sigma) were prepared in phosphate-buffered saline (PBS) buffer, pH 6.8, adjusted to an ionic strength *I* = 0.1 M by the addition of NaCl, according to Green, 1993. Deacetylated chitosan was also purchased from Sigma Aldrich (Kitozyme, 740,179-5 g, Dorset, UK). The carboxymethylcellulose (CMC) and pullulan samples were purchased from Carbosynth, UK. DMAE-pullulan was obtained as described previously [30]. Briefly, 5 g of pullulan was dissolved in 25 ml of distilled water and mixed with a 25 mL 10 M sodium hydroxide solution. Then, 35 g of 2-chloro-N,N dimethylethylamine hydrochloride was added to the mixture and left stirring at 60 °C for 1 h. After the reaction was completed, the mixture was washed four times with 50 ml diethyl ether and after was diluted in water to a concentration of 10 mgmL^−1^ and adjusted to pH 7 using HCl. The solution was further cleaned of organic solvents and concentrated in a rotary evaporator, after which was dialysed in PBS buffer on a 14,000 Da (g/mol) membrane for two days. The resulting solution was freeze-dried which resulted in the formation of white odourless powder. The powder was stored at 4 °C until needed.

### 4.2 Mucin beads preparation

A 10 mg/mL pig gastric mucin solution and was prepared in 0.1 M PBS (pH 6.8) and mixed with a 40 mg/mL Na^+^ alginate aqueous solution. The final concentrations of mucin and alginate were 5 mg/mL and 20 mg/mL, respectively. A syringe was used to pour even droplets into a 3% calcium chloride solution, and left to harden for 30 min under constant stirring. The control beads (Na^+^ alginate) were prepared in the same way, such that the final concentration was 20 mg/mL, but without the presence of mucin. The beads were collected and hydrated in RO water (reverse osmosis) prior to investigation.

### 4.3 Orange drink preparation

Robinson's orange squash was used throughout this investigation, purchased from the local supermarket. The original formulation was additionally infused with hexanal, octanal and decanal, such that their final concentration was 1 ppm (1 mg/L). Then, 2 mg/mL stock solutions of CMC, pullulan and DMAE-pullulan were prepared in 0.1 M PBS (pH 6.8) while sodium acetate buffer (pH 5) was used to solubilise chitosan. Final solutions were made such that they contain one part orange squash, one part polymer solution, and three parts water. The final polymer concentrations were 0.4 mg/mL, which are below the critical coil overlap concentration c*, in order to reduce viscosity related effects [33].

### 4.4 Titratable acidity

Five milliliters of the final solution weas mixed with 25 mL distilled water. A 0.1 N NaOH solution was constantly added in using a 200 μl Gilson's pipette, until the solution reached a pH of ~8. The volume of NaOH solution added was recorded and used to determine the titratable acidity of each sample using the following equation:

(1) $$\mathrm{TA}=\left(\mathrm{Vol}\;\text{of NaOH}\right)\times \left(N\;\text{of NaOH}\right)\times \left(\mathrm{Eq}.\text{factor of acid}\right)/\left(\mathrm{Wt}.\text{of sample}\times 1000\right)\times 100$$

### 4.5 *Ex-vivo* tissue preparation

**Fig. 7:**
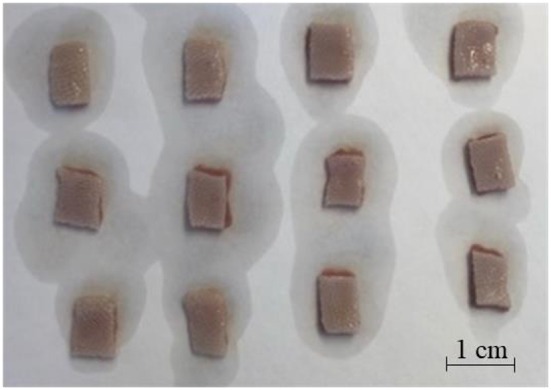
The top layer of the bovine tongue cut into rectangles prior to being treated with different biopolymer solutions.

A freshly cut ox tongue was delivered up to 24 h post slaughter. Most of the muscle and connective tissues were immediately separated from the upper surface of the tongue, and refrigerated overnight. Freezing was avoided to reduce potential undesired damage to the oral mucosa (see Hegarty 1973). The dorsal surface of the tongue was gently separated from the remaining muscle tissue using surgical blades and shaped into equal sized rectangles (1 × 1.5 cm) of around 2 mm in thickness (Fig. 7).

The tip and back part of the tongue were not used to avoid discrepancies arising from their different morphologies, which contain large or an uneven number of very ubiquitous papillae. The sections were submerged in the different orange drink formulations for approximately 5 min and placed into GC vials for headspace analysis.

### 4.6 Retention analysis and weight experiments

The weight of five control and five mucin beads were recorded before and after immersion in the model drink containing the different polymer solutions. For the GC-analysis, vials containing the mucin beads were placed on a roller mixer for approximately 5 min. The beads were drained on a 250 μm sieve to remove excess liquid and were sealed in GC-vials containing 1 mL of water to maintain hydration. The experiment was performed in triplicate.

### 4.7 Conductivity meter

Changes in the intensity of sodium ions were evaluated using a Mettler Toledo conductivity meter (Ohio, USA). The experimental design was the same as described for the GC analysis. The only difference was that the beads were immersed final solutions contained 1% (w/v) NaCl. The beads were immersed in 15 mL RO water, prior to analysis and allowed to rest for approximately 20 min. The change in conductivity is directly proportional to the amount of Na^+^ ions retained into the bead.

### 4.8 Gas chromatography-mass spectrometry (GC–MS)

The Trace 1300 series Gas Chromatograph coupled with the single-quadrupole mass spectrometer (Thermo Fisher Scientific, Hemel Hempstead, UK) was used. Samples were incubated at 37.0 °C for 20 min with intermittent stirring. Then, the solid phase microextraction (SPME) fiber (50/30 μm DVB/CAR/PDMS, Supelco, Sigma Aldrich, UK) was used to extract for 40 min then desorb for 1 min. Separation was carried out by a ZB-WAX capillary gas chromatography column (length 30 m, internal diameter 1 mm, 1.00 μm film thickness). The column temperature was initially at 40.0 °C for 2 min, then increased by 6.0 °C every minute up until 250.0 °C and held for 5 min. Full scan mode was chosen to measure volatile compounds (mass range from 20 to 300 Da). A splitless mode was used, and a constant carrier pressure of 18 psi was applied. Volatiles were identified by comparison of each mass spectrum with the spectra from the *NIST* Mass Spectral Library.

### 4.9 Dynamic light scattering (DLS)

The experiments were performed using the Zetasizer Nano-ZS detector and low volume disposable sizing cuvettes (Malvern Instruments Ltd., Malvern, UK). The samples were measured at (20.00 ± 0.01) °C using the dip-cell as instructed by the manufacturer (Malvern Instruments Ltd., Malvern, UK). The bead is held in place between the two electrodes and placed in a standard cuvette (ZEN0112). An applied voltage of 2 mV was used and the apparent electrophoretic mobility of the bead was measured. It was assumed that the sample does not move during the experiment, therefore no glue was used in the attachment of the beads between the two electrodes. The surface zeta potential cell SOP was employed in automatic mode and repeated 6 times.

### 4.10 Environmental scanning electron microscopy (ESEM)

Mucin beads were also analysed using a Thermofisher Scientific (Waltham, USA) FEI Quanta 650 ESEM. Samples were cooled to 2.0 °C by means of a Peltier cooling stage, and the pressure of water vapour in the chamber was adjusted to maintain a relative humidity of between 60 and 90%. An accelerating voltage of 15 kV was used for all samples.

### 4.11 Analytical ultracentrifugation- sedimentation velocity (AUC-SV)

SV experiments were performed at 20.0 °C using the Optima XL-I analytical ultracentrifuge (Beckman, Palo Alto, USA) equipped with Rayleigh interference optics. A volume of 395 μl sample and 405 μl solvent respectively were injected into 12 mm double sector epoxy cells with sapphire windows and centrifuged at 30000 rpm. The cells were balanced to 0.01 g. The interference and absorbance systems were employed to record changes in concentration *versus* radial displacement. Data was analysed in SEDFIT using the *g*(s) method of Dam and Schuck (2003) [31,32] by generating sedimentation coefficient distributions, *g*(s) *vs* s, where s is the sedimentation coefficient in Svedberg units, S = 10^−13^ s. TI and RI noise was removed before fitting the data.

### 4.12 Statistical analysis

GC–MS and conductivity samples were randomised and the analysis was made using Tukey's *post hoc* test to identify significance (*p* < 0.05 expressed as *). Figures were made in Origin 7.5 (OriginLab, Massachusetts, USA).
